# Incidence and multidimensional predictors of occasional and recurrent falls among Malaysian community‐dwelling older persons

**DOI:** 10.1186/s12877-021-02103-2

**Published:** 2021-03-02

**Authors:** Theng Choon Ooi, Devinder Kaur Ajit Singh, Suzana Shahar, Nor Fadilah Rajab, Divya Vanoh, Razinah Sharif, Maw Pin Tan

**Affiliations:** 1 Centre for Healthy Ageing and Wellness, Faculty of Health Sciences, Universiti Kebangsaan Malaysia, Jalan Raja Muda Abdul Aziz, 50300 Kuala Lumpur, Malaysia; 2 Nutrition & Dietetics Programme, School of Health Sciences, Health Campus, Universiti Sains Malaysia, 16100 Kubang Kerian, Kelantan Malaysia; 3 Department of Medicine, Faculty of Medicine, University of Malaya, 50603 Kuala Lumpur, Malaysia; 4 Ageing and Age-Associated Disorders Research Group, Faculty of Medicine, University of Malaya, 50603 Kuala Lumpur, Malaysia

**Keywords:** Depression, Falls, Hemoglobin, Incidence, Muscle strength, Older persons, Predictors

## Abstract

**Background:**

Falls incidence rate and comprehensive data on factors that predict occasional and repeated falls from large population-based studies are scarce. In this study, we aimed to determine the incidence of falls and identify predictors of occasional and recurrent falls. This was done in the social, medical, physical, nutritional, biochemical, cognitive dimensions among community-dwelling older Malaysians.

**Methods:**

Data from 1,763 Malaysian community-dwelling older persons aged ≥ 60 years were obtained from the LRGS-TUA longitudinal study. Participants were categorized into three groups according to the presence of a single fall (occasional fallers), ≥two falls (recurrent fallers), or absence of falls (non-fallers) at an 18-month follow-up.

**Results:**

Three hundred and nine (17.53 %) participants reported fall occurrences at an 18-month follow-up, of whom 85 (27.51 %) had two or more falls. The incidence rate for occasional and recurrent falls was 8.47 and 3.21 per 100 person-years, respectively. Following multifactorial adjustments, being female (OR: 1.57; 95 % CI: 1.04–2.36), being single (OR: 5.31; 95 % CI: 3.36–37.48), having history of fall (OR: 1.86; 95 % CI: 1.19–2.92) higher depression scale score (OR: 1.10; 95 % CI: 1.02–1.20), lower hemoglobin levels (OR: 0.90; 95 % CI: 0.81-1.00) and lower chair stand test score (OR: 0.93; 95 % CI: 0.87-1.00) remained independent predictors of occasional falls. While, having history of falls (OR: 2.74; 95 % CI: 1.45–5.19), being a stroke survivor (OR: 8.57; 95 % CI: 2.12–34.65), higher percentage of body fat (OR: 1.04; 95 % CI: 1.01–1.08) and lower chair stand test score (OR: 0.87; 95 % CI: 0.77–0.97) appeared as recurrent falls predictors.

**Conclusions:**

Having history of falls and lower muscle strength were predictors for both occasional and recurrent falls among Malaysian community-dwelling older persons. Modifying these predictors may be beneficial in falls prevention and management strategies among older persons.

## Background

The global prevalence of falls was 5,186 (4,622–5,849) per 100,000 people in year 2017 [1]. Falls can lead to debilitating consequences, including injury-related hospitalization, disability and death [2]. Moreover, fall-related injuries in older persons result in increased healthcare costs. The estimated medical costs of fatal and non-fatal falls among older persons in the US was reported to be around 50 billion dollars [3]. Population-wide falls prevention programs that effectively improve the overall health and reduce social care burden associated with fall-related complications are, therefore, urgently required [4].

Several risk factors have been identified to be associated with falls in older persons. Females and those who are older in age are at higher risk of falls [5]. In the psychosocial context, older persons who live alone, have fewer years of education, or who experience loneliness and depression are more likely to be at risk of falls [6]. Medical illnesses such as arthritis, diabetes, chronic kidney diseases and stroke are among the other risk factors for falls in older persons [6, 7]. Physical frailty, cognitive decline and other geriatric syndromes also contribute to the occurrence of falls in older persons [5].

Declined physical function below a certain threshold, manifesting as muscle weakness and loss of balance are likely to lead to falls in older persons as well [8]. In addition, older persons with cognitive impairment are twice more likely to fall in comparison to those without cognitive impairment [9]. Nutritional deficiencies, such as vitamin D, calcium and inadequate protein intake are linked to osteoporosis, fractures and sarcopenia and, as a result, increase both the risk of falls and its consequences [10]. Environmental factors, for example, poor lighting, clutter in and around the house and inadequate footwear are often contributors to the risk of falls among older persons [5].

It is noteworthy that most risk factors of falls studied among community-dwelling older persons were performed via cross-sectional studies and were inconclusive. In addition, physical, nutritional, biochemical and biomarker status were not examined together within a single study. As falls are multifactorial in nature, identifying the multidimensional predictors of falls risk may be beneficial for early falls prevention strategies in community-dwelling older persons. The objective of this study was to determine the incidence of falls and identify predictors of occasional and recurrent falls. This was done in the social, medical, physical, nutritional, biochemical and cognitive dimensions among community-dwelling older Malaysians.

## Methods

### Participants

This prospective cohort study involved 1,763 participants from the first wave of the Long-term Research Grant Scheme - Towards Useful Ageing (LRGS-TUA) study who were successfully followed up at 18-months (wave 2). Participants were recruited from four states representing Malaysia’s northern, central, southern and east coast zones through a multistage random sampling approach, as reported by Shahar et al. (2016) [11]. Older persons aged 60 years and above, able to walk with or without aids and those who were able to converse in Malay, English, Chinese, or Tamil language participated in the study. Older persons with severe visual, hearing, musculoskeletal or neurological impairments were excluded. This study was approved by the Medical Research and Ethics Committee of Universiti Kebangsaan Malaysia (UKM 1.5.3.5/244/NN-060-2013).

### Data collection

In wave 1, demographic data and several risk factors were assessed as described in the previous study [11]. These assessments are described briefly below:

#### Demographic data

A standardized questionnaire was used to obtain the socio-demographic and health data from the participants, including age, sex, ethnicity, marital status, living status, years of education, smoking habits, alcohol drinking status and self-reported medical history.

#### Body composition and blood pressure

The height, weight and the circumference of the waist, hip and calf were measured using a SECA 206 portable body meter (Seca, Hamburg, Germany), Tanita digital lithium weighing scale (Tanita, Tokyo, Japan) and Lufkin tape respectively, as reported earlier [11]. The body mass index (BMI) of participants were then calculated by using the formula “body weight (kg)/height (m)^2^. The fat-free mass, fat mass, skeletal muscle mass and percentage of body fat were measured by using the InBody S10 body composition analyzer (Biospace Co. Ltd, Korea). Meanwhile, the systolic and diastolic blood pressure was taken twice using an automatic digital blood pressure monitor (OMRON, Kyoto, Japan) to obtain the average reading.

#### Dietary Status

Participants were interviewed using a validated Dietary History Questionnaire for older persons to record their usual dietary intake in a week, as previously described [11]. The nutrient intake was analyzed by using Nutritionist Pro software.

#### Biochemical analysis

Fasting venous blood was collected from the participants by a trained phlebotomist and subjected to biochemical analysis. The parameters included in the analysis were fasting blood glucose, serum albumin, hemoglobin levels and serum lipid profile [total cholesterol, high-density lipoprotein (HDL), low-density lipoprotein (LDL) and triglyceride levels].

#### Cognitive status

The following tests were performed:

i. Mini-Mental State Examination

General cognitive function was assessed using the Malay version of the Mini-Mental State Examination (MMSE), which is a valid and reliable screening tool for dementia in the Malaysian population [12].

ii. Digit Span

Digit Span [subtest for the Wechsler Adult Intelligence Scale (WAIS)] is a sensitive tool used to measure working memory [13]. This test is used to assess attention.

iii. Montreal Cognitive Assessment

The Malay version of the Montreal Cognitive Assessment (MoCA) [14] was used to test for orientation, short memory recall, attention and concentration, working memory and mental arithmetic, language comprehension, visuospatial domain, executive cognitive functioning, naming of objects and abstract thought components.

iv. Digit symbol

Digit symbol (a subset of WAIS) was used to evaluate visual-motor speed and coordination, visual search and cognitive flexibility [15]. This test requires participants to fill in small blank squares with symbols that matched the number labeled in each square. It is one of the most age-sensitive and useful cognitive tests to distinguish between mild Alzheimer-type dementia and healthy ageing.

#### Psychosocial

The validated Malay version of the Geriatric Depression Scale-15 [16] was used to evaluate depression status.

#### Physical Performance

i. Timed Up and Go test

In the Timed Up and Go (TUG) test, participants were required to sit on an armless chair with a seating height of 46 cm. Participants were then instructed to stand up, walk 3 meters at a normal pace, turn around, walk back and sit on the chair. The TUG test was performed twice consecutively and the average time (seconds) to complete the test was recorded. The participants were allowed to walk with their regular walking aids.

ii. 2-minute step test

The 2-minute step test measures aerobic endurance. Participants were instructed to march on the spot to about 90° hip flexion and 90° knee extension. The test was continued for 2 minutes and the number of steps taken were counted.

iii. 30-second chair stand test

Participants were required to sit on a chair (without armrest) with hands on opposite shoulders crossed at the wrists. They were then instructed to rise to full standing and sitting down again and to repeat this task for 30 seconds. The number of times the sit-to-stand were recorded. This was done twice, with a rest in between. This test reflects lower limb muscle strength.

iv. Dominant handgrip muscle strength test

The dominant handgrip muscle strength test was performed using a handgrip dynamometer (Jamar Plus+ Hand Dynamometer, SI Instruments Pty Ltd, SA, Australia). The maximum effort was measured twice with shoulder in sustained adduction and neutral rotation, elbow flexed at 90 degrees, forearm neutral and wrist between 0-15 degrees of ulnar deviation. The highest reading was taken as the result.

#### Falls history at Baseline and follow‐up

During the first wave of the study, participants were asked, “Have you had a fall in the past 12-months?”. At the 18-month follow-up interview, participants were asked, “Have you had a fall in the past 18-months?”. If they responded positively to the above question, a further question, “How many times have you fallen?” were asked. The participants were informed that a fall is an event whereby a person is ‘inadvertently coming to rest on the ground, floor or other lower levels, excluding intentional change in position to rest on furniture or floor [17]. Participants who reported to have no fall history in the past during follow-up were categorized as non-fallers. Those with one fall were categorized as occasional fallers, while those who reported two or more falls were categorized as recurrent fallers.

### Statistical analysis

The cumulative incidence of falls was calculated by dividing the number of new cases of a fall or recurrent falls at 18-months follow-up with the total number of participants that were successfully followed up at wave 2 of the study. The incidence rate of falls was calculated by using the person-years method, whereby the number of new cases of a fall or recurrent falls between the two data collection points was computed to the number of cases per 100 person-years. The age-specific incidence rate was then calculated by further dividing the participants into four different age-groups (60–64, 65–69, 70–74 and 75 years and above).

Socio-demographic data, body composition, nutrition, biochemical, cognitive and physical performance scores were compared between non-fallers and occasional fallers, as well as non-fallers and recurrent fallers by using the independent t-test for continuous variables or Chi-square test for categorical variables. Variables that were found to be significant (*p* < 0.05) in the univariate tests were further analyzed using binary logistic regression analyses. These were then entered into the final multivariate logistic regression analysis using the forward-stepwise logistic model to determine falls predictors. Confounding factors (age, sex, multimorbidity, falls history at baseline, years of education and cognitive performance) were included for adjustment in the multivariate logistic regression model. The statistical analyses were performed using Statistical Package for the Social Sciences (SPSS) version 25 (IBM Corp., Armonk, NY).

## Results

Three hundred and nine (17.53 %) participants reported fall occurrence at an 18-month follow-up, of whom 85 (27.51 %) had two or more falls. The incidence rate for occasional and recurrent falls was 8.47 and 3.21 per 100 person-years, respectively. The age-specific incidence rates of both occasional and recurrent falls are as shown in Table 1. Overall, the incidence rate of falls was independent of increasing age. The ≥ 75 age group had the highest incidence rate for occasional falls (9.09 per 100 person-years). While the 65–69 years age group had the highest incidence rate for recurrent falls (3.91 per 100 person-years).

**Table 1:** The age-specific incidence rates of occasional falls and recurrent falls over 18-months

Age group	No. of Participants	Occasional falls			Recurrent falls		
		No. of cases	Cases/year	Cases/100 person-years	No. of cases	Cases/year	Cases/100 person-years
60–64	523	64	42.67	8.16	24	16.00	3.06
65–69	528	68	45.33	8.59	31	20.67	3.91
70–74	404	50	33.33	8.25	14	9.33	2.31
≥ 75	308	42	28.00	9.09	16	10.67	3.46
Total	1,763	224	149.33	8.47	85	56.67	3.21

Table 2 depicts the baseline characteristics of non, occasional and recurrent fallers. Occasional fallers were more likely (*p* < 0.05) to be women (60.27 %), single (3.13 %), have falls history (30.36 %), have higher depression scale scores (2.95 ± 2.42); lower fat-free mass (35.64 ± 7.07 kg) and skeletal muscle mass (18.82 ± 4.21 kg); lower intake of energy (1,583.99 ± 436.79 kcal/day), carbohydrate (209.95 ± 67.82 g/day) and zinc (3.38 ± 1.60 mg/day); lower hemoglobin levels (13.64 ± 1.85 g/L), scored lower in MMSE (22.62 ± 4.82), MoCA (18.35 ± 5.82) and digit symbol (4.73 ± 2.50); scored lower in chair stand (9.60 ± 3.28 times) and dominant handgrip muscle strength (20.82 ± 7.27 kg) tests. Recurrent fallers were more likely (*p* < 0.05) to be women (68.24 %), living alone (17.65 %), have falls history (42.35 %), with a medical history of stroke (4.71 %) and joint pain (36.47 %); higher depression scale score (3.35 ± 2.63); lower fat-free mass (35.17 ± 7.90 kg) and skeletal muscle mass (18.51 ± 4.70 kg); higher fat mass (26.32 ± 10.16 kg) and percentage of body fat (41.89 ± 10.26 %); lower hemoglobin levels (13.42 ± 2.13 g/L); scored lower in chair stand (8.83 ± 3.07 times), TUG (12.84 ± 5.28 seconds) and dominant handgrip muscle strength (19.57 ± 6.69 kg) tests.

**Table 2:** The baseline attributes of the participants with no fall, occasional and recurrent falls

Parameters	Non-fallers (*n* = 1454) [Prevalence (%) or mean ± SD]	Occasional fallers (*n* = 224) [Prevalence (%) or mean ± SD]	*P*-value	Recurrent fallers (*n* = 85) [Prevalence (%) or mean ± SD]	*P*-value
**Age (years)**	68.62 ± 5.94	68.80 ± 6.13	0.668	68.65 ±6.07	0.967
**Sex**					
Male	751 (51.65)	89 (39.73)	0.001*	27 (31.76)	<0.001*
Female	703 (48.35)	135 (60.27)		58 (68.24)	
**Ethnicity**					
Malay	879 (60.45)	146 (65.18)	0.119	58 (68.24)	0.218
Chinese	509 (35.01)	64 (28.57)		22 (25.88)	
Indian	66 (4.54)	14 (6.25)		5 (5.88)	
**Single**	18 (1.24)	7 (3.13)	0.030*	1 (1.18)	0.960
**Living alone**	138 (9.49)	22 (9.82)	0.875	15 (17.65)	0.015*
**Smoking**	251 (17.26)	34 (15.18)	0.439	10 (11.76)	0.189
**Alcohol consumption**	66 (4.54)	7 (3.13)	0.334	2 (2.35)	0.340
**Education (years)**	5.38 ± 4.03	4.88 ± 3.79	0.083	4.54 ± 4.04	0.063
**Falls history**	222 (15.27)	68 (30.36)	<0.001*	36 (42.35)	<0.001*
**Chronic diseases**					
Hypertension	714 (49.11)	116 (51.79)	0.455	46 (54.12)	0.369
Diabetes mellitus	363 (24.97)	57 (25.45)	0.877	24 (28.24)	0.499
Stroke	22 (1.51)	3 (1.34)	0.842	4 (4.71)	0.026*
Joint Pain	337 (23.18)	64 (28.57)	0.078	31 (36.47)	0.005*
Cardiovascular diseases	147 (10.11)	21 (9.38)	0.733	7 (8.24)	0.576
Cataract & glaucoma	127 (8.73)	27 (12.05)	0.109	7 (8.24)	0.874
Asthma	111 (7.63)	18 (8.04)	0.834	10 (11.76)	0.169
Gout	68 (4.68)	7 (3.13)	0.295	5 (5.88)	0.611
Gastric ulcer	178 (12.24)	34 (15.18)	0.218	15 (17.65)	0.144
Urinary Incontinence	143 (9.83)	26 (11.61)	0.412	11 (12.94)	0.354
Hearing & vision problems	173 (11.90)	23 (10.27)	0.479	15 (17.65)	0.116
**Psychosocial**					
Depression	2.54 ± 2.19	2.95 ± 2.42	0.012*	3.35 ± 2.63	0.007*
**Physical**					
BMI (kg/m^2^)	24.95 ± 4.41	25.11 ± 4.83	0.606	25.68 ± 5.07	0.140
Circumference: waist (cm)	88.21 ± 11.20	88.08 ± 11.97	0.870	89.88 ± 12.62	0.239
Circumference: hip (cm)	96.54 ± 9.18	96.95 ± 10.37	0.575	98.46 ± 11.61	0.139
Circumference: calf (cm)	33.55 ± 3.75	33.12 ± 3.85	0.114	33.41 ± 4.08	0.744
Fat mass (kg)	24.19 ± 9.09	24.47 ± 9.67	0.680	26.32 ± 10.16	0.038*
Fat free mass (kg)	37.16 ± 7.89	35.64 ± 7.07	0.004*	35.17 ± 7.90	0.025*
Skeletal muscle mass (kg)	19.75 ± 4.71	18.82 ± 4.21	0.003*	18.51 ± 4.70	0.019*
Percentage of body fat (%)	38.68 ± 10.38	39.69 ± 10.20	0.178	41.89 ± 10.26	0.006*
Systolic (mmHg)	140.70 ± 22.40	141.13 ± 20.34	0.795	138.81 ± 23.46	0.471
Diastolic (mmHg)	77.26 ± 13.44	76.87 ± 12.96	0.689	77.31 ± 12.88	0.975
**Nutrition**					
Energy (kcal/day)	1,658.03 ± 485.42	1,583.99 ± 436.79	0.037*	1,625.96 ± 509.31	0.560
Protein (g/day)	70.85 ± 22.10	68.75 ± 23.29	0.201	69.36 ± 22.77	0.550
Carbohydrate (g/day)	224.76 ± 77.79	209.95 ± 67.82	0.004*	218.05 ± 81.67	0.447
Sugar (g/day)	21.51 ± 15.12	20.42 ±14.94	0.331	18.51 ± 12.94	0.077
Fat (g/day)	52.87 ± 20.69	51.79 ± 20.36	0.477	53.13 ± 20.72	0.912
Cholesterol (mg/day)	158.51 ± 113.21	162.88 ± 122.33	0.606	172.06 ± 130.20	0.294
Saturated fat (mg/day)	8.39 ± 5.73	8.20 ± 6.14	0.681	9.05±6.10	0.307
MUFA (g/day)	8.32 ± 5.02	8.44 ± 5.65	0.750	8.71 ± 5.73	0.498
PUFA (g/day)	5.44 ± 3.43	5.49 ± 3.67	0.834	5.40 ± 3.22	0.916
Vitamin D (mg/day)	0.35 ± 2.50	0.27 ± 0.97	0.682	0.34 ± 1.05	0.990
Vitamin E (mg/day)	12.04 ± 62.58	14.77 ± 71.91	0.563	4.33 ± 2.40	0.262
α-tocopherol (mg/day)	0.45 ± 1.17	0.51 ± 1.45	0.506	0.54 ± 1.22	0.495
Sodium (mg/day)	1,466.23 ± 979.55	1,401.85 ± 1068.97	0.545	1,466.36 ± 799.12	0.854
Potassium (mg/day)	1,510.35 ± 552.18	1,443.59 ± 522.91	0.099	1,402.60 ± 534.01	0.083
Calcium (mg/day)	520.70 ± 248.06	501.46 ± 242.21	0.292	473.34 ± 212.43	0.089
Iron (mg/day)	13.51 ± 5.41	12.96 ± 4.84	0.160	13.26 ± 6.10	0.681
Phosphorus (mg/day)	1,094.54 ± 418.46	1,081.38 ± 425.24	0.671	1,067.02 ± 389.09	0.559
Magnesium (mg/day)	131.86 ± 64.94	128.51 ± 68.80	0.489	123.00 ± 63.66	0.227
Zinc (mg/day)	3.63 ± 1.99	3.38 ± 1.60	0.047*	3.40 ± 1.65	0.310
Selenium (μg/day)	23.98 ± 18.23	22.47 ± 17.17	0.260	23.88 ± 16.95	0.961
**Biochemical**					
Hemoglobin (g/L)	14.20 ± 2.30	13.64 ± 1.85	<0.001*	13.42 ± 2.13	0.008*
Glucose (mmol/L)	6.19 ± 2.28	5.95 ± 1.69	0.096	6.18 ± 2.00	0.987
Cholesterol (mmol/L)	5.41 ± 1.12	5.47 ± 1.09	0.532	5.54 ± 1.05	0.359
HDL (mmol/L)	1.40 ± 0.34	1.41 ± 0.39	0.726	1.47 ± 0.46	0.121
LDL (mmol/L)	3.34 ± 1.03	3.36 ± 0.95	0.806	3.42 ± 0.98	0.523
Triglyceride (mmol/L)	1.50 ± 0.76	1.55 ± 0.76	0.513	1.45 ± 0.63	0.598
Albumin (g/L)	42.93 ± 2.77	42.86 ± 2.75	0.736	42.42 ± 2.90	0.158
**Cognitive Test**					
Digit Span	7.62 ± 2.42	7.46 ± 2.37	0.346	7.43 ± 2.40	0.479
MMSE	23.32 ± 4.64	22.62 ± 4.82	0.037*	22.77 ± 5.16	0.299
MoCA	19.23 ± 5.57	18.35 ± 5.82	0.031*	18.56 ± 5.88	0.294
Digit Symbol	5.14 ± 2.59	4.73 ± 2.50	0.038*	4.55 ± 2.29	0.059
**Physical performance **					
2-minute step test (number)	62.44 ± 25.45	60.70 ± 24.96	0.350	56.31 ± 32.72	0.104
Chair stand test (number)	10.05 ± 2.98	9.60 ± 3.28	0.039*	8.83 ± 3.07	<0.001*
Timed Up and Go test (seconds)	11.33 ± 3.56	11.62 ± 3.49	0.258	12.84 ± 5.28	0.013*
Dominant handgrip muscle strength test (kg)	22.59 ± 7.66	20.82 ± 7.27	0.002*	19.57 ± 6.69	0.001*

*Note*: *BMI *Body mass index, *HDL* High-density lipoprotein, *LDL* Low-density lipoprotein, *MMSE *Mini-mental state examination, *MoCA* Montreal cognitive assessment, *MUFA* Monounsaturated fatty acids, *PUFA* Polyunsaturated fatty acids. *Significant differences as compared to non-fallers (*p*<0.05)

Variables that were significant in the univariate test were entered into binary logistic regression. The univariate predictors of occasional and recurrent falls are as shown in Table 3. These variables were then further analyzed using multivariate logistic regression analysis (Table 4). Being female (OR: 1.57; 95 % CI: 1.04–2.36), being single (OR: 5.31; 95 % CI: 3.36–37.48), having history of falls (OR: 1.86; 95 % CI: 1.19–2.92) higher depression scale scores (OR: 1.10; 95 % CI: 1.02–1.20), lower hemoglobin levels (OR: 0.90; 95 % CI: 0.81-1.00) and lower chair stand test scores (OR: 0.93; 95 % CI: 0.867-1.00) appeared as occasional falls predictors in this model [χ² (df = 8, N = 1763) = 13.38, *p* = 0.100 with 86.60 % accuracy]. While, having history of falls (OR: 2.74; 95 % CI: 1.45–5.19), stroke (OR: 8.57; 95 % CI: 2.12–34.65), higher percentage of body fat (OR: 1.04; 95 % CI: 1.01–1.08) and lower scores in chair stand test (OR: 0.87; 95 % CI: 0.77–0.97) were identified as predictors of recurrent falls [χ² (df = 8, N = 1763) = 10.82, *p* = 0.212 with 94.70 % accuracy].

**Table 3:** Univariate scores for individual predictors of occasional and recurrent falls

Domains	Variables	*P*-value	Exp(B)	[95 % CI]
**Occasional falls**				
Sociodemographic	Sex (Female)	0.001*	1.62	1.22–2.16
	Marital status (Single)	0.036*	2.57	1.06–6.23
	Falls history	< 0.001*	2.42	1.76–3.33
Psychosocial	Depressive symptoms	0.013*	1.08	1.02–1.14
Body composition	Fat-free mass	0.007*	0.97	0.96–0.99
	Skeletal muscle mass	0.006*	0.96	0.93–0.99
Nutrition	Energy	0.037*	1.00	1.00–1.00
	Carbohydrate	0.009*	1.00	1.00–1.00
	Zinc	0.089*	0.93	0.86–1.01
Biochemical	Hemoglobin	0.002*	0.88	0.88–0.96
Cognitive test	MMSE	0.037*	0.97	0.94-1.00
	MoCA	0.031*	0.97	0.95-1.00
	Digit Symbol	0.039*	0.94	0.88-1.00
Physical performance	Chair stand test	0.039*	0.95	0.91-1.00
	Dominant handgrip muscle strength test	0.002*	0.97	0.95–0.99
**Recurrent falls**				
Sociodemographic	Sex (Female)	0.001*	2.30	1.44–3.67
	Living alone	0.017*	2.04	1.14–3.67
	Falls history	< 0.001*	4.08	2.59–6.42
Chronic disease	Stroke	0.036*	3.21	1.08–9.55
	Joint pain	0.006*	1.90	1.20–3.01
Psychosocial	Depressive symptoms	0.001*	1.14	1.05–1.24
Body composition	Fat mass	0.039*	1.03	1.00-1.05
	Fat-free mass	0.025*	0.97	0.94-1.00
	Skeletal muscle mass	0.020*	0.94	0.90–0.99
	Percentage of body fat	0.006*	1.03	1.01–1.05
Biochemical	Hemoglobin	0.008*	0.83	0.72–0.95
Physical performance	Chair stand test	< 0.001*	0.87	0.80–0.94
	Timed Up and Go test	0.001*	1.08	1.03–1.13
	Dominant handgrip muscle strength test	0.001*	0.95	0.92–0.98

*Note*: *MMSE *Mini-mental state examination, *MoCA* Montreal cognitive assessment*p<0.05

**Table 4:** Independent predictors for occasional and recurrent falls at 18 months follow-up

Domains	Variables	*P*-value	Exp(B)	[95 % CI]
**Occasional falls**				
Sociodemographic	Sex (Female)	0.033*	1.57	1.04–2.36
	Marital status (Single)	< 0.001*	5.31	3.36–37.48
	Falls history	0.006*	1.86	1.19–2.92
Psychosocial	Depressive symptoms	0.014*	1.10	1.02–1.20
Biochemical	Hemoglobin	0.040*	0.90	0.81-1.00
Physical performance	Chair stand test	0.040*	0.93	0.87-1.00
**Recurrent falls**				
Sociodemographic	Falls history	0.002*	2.74	1.45–5.19
Chronic disease	Stroke	0.003*	8.57	2.12–34.65
Body composition	Percentage of body fat	0.011*	1.04	1.01–1.08
Physical performance	Chair stand test	<0.001*	0.87	0.77–0.97

*Note*: *p<0.05

## Discussion

We identified the incidence rate and multidimensional falls risk predictors at 18-months follow-up among 1,763 community-dwelling older Malaysians. The incidence rate of occasional and recurrent falls observed in our study was 8.47 and 3.21 per 100 person-years respectively, with no increase with age. Falls prevalence of 15–18 % [18, 19] and 27 % over a six-months follow-up period [20] had previously been reported among community-dwelling older Malaysians. The prevalence of recurrent falls is reported as 8.3 % [21].

Despite the apparent association between age and decline in both physical and cognitive functions [5], our results showed that advancing age did not predict the incidence of occasional and recurrent falls. In an age-specific population, the incidence of falls was not age-dependent, as opposed to the prevalence of falls [1]. This suggests that the number of falls does not increase with increasing age. It may be possible that cumulatively, the number of falls among older people in any given period of time could be observed due to the consistent addition of new cases. Decline in memory with age might also be another reason that may have affected the ability of older persons to recall falls incidences.

Occasional falls could be accidental and are usually associated with extrinsic factors [22, 23]. In comparison, recurrent falls among older persons are commonly related to multifactorial intrinsic factors suggesting a more complex risk model. Our study findings showed that the identified predictors for both occasional and recurrent falls were different, with the exception of having history of falls and taking longer to complete the chair stand test. Having lower muscle strength and experiencing falls in the past appeared as robust predictors of both occasional and recurrent falls in community-dwelling older persons. Being female, single, having higher depression scale score and lower hemoglobin levels were predictors of occasional falls. While, predictors of recurrent falls included a history of stroke and having higher percentage of body fat.

A history of falls appeared to be the predictor of both occasional and recurrent falls. These findings are consistent with previous reports where having a history of falls was found to be a significant predictor of subsequent falls [24]. Furthermore, our study results showed that older women had higher risk of falls as compared to men. Older women having a higher risk of falls has been well established in the literature. Previously, the English Longitudinal Study of Ageing (ELSA) involving 1994 men and 2357 women had reported higher prevalence of falls among women (29.1 %) compared to men (23.5 %) [2]. The number of women admitted to hospital due to falls increased every year from 15,000 in year 2001 to 20,000 in 2009 [25]. It is also plausible that women have a greater loss in bone mineral density due to menopause and this could be associated with decline in muscle strength and falls.

Depression is associated with occasional falls among community-dwelling older persons as reported previously [26]. Depressive symptoms may affect older persons’ mobility and executive function [27]. The causal relationship between depression and falls was not fully explained by adjustment for the medical comorbidities, nutritional, physical and biochemical factors. Use of medications was not adjusted within this study, which may account for the increased risk of falls among individuals with symptoms of depression [28]. Depression in older persons has been attributed to structural brain changes that interfere with cortical-subcortical circuits, basal-ganglia and limbic networks, which in turn affect postural stability leading to the occurrence of falls [29]. Furthermore, antidepressants have been associated with single and recurrent falls [29, 30]. The proposed mechanisms for antidepressant-related falls include orthostatic hypotension, dizziness, compromised vision and mental confusion [30].

Lower hemoglobin levels increased the risk of occasional falls. Potential mechanisms linking the age-associated decline in hemoglobin and falls include fatigue, reduced muscle strength and muscle quality. The decline in oxygen delivery is attributed to the reduction of hemoglobin levels, whereby hemoglobin functions as an oxygen carrier to skeletal muscles, leading to a reduction in muscle function and declining mobility. This finding was in agreement with the three-year Longitudinal Aging Study Amsterdam demonstrating frequent episodes of falls among older persons with anemia as compared to their non-anemic counterparts [31]. The presence of lower hemoglobin may also reflect underlying nutritional deficiencies or chronic conditions affecting hemoglobin production. Others include undetected causes of hemoglobin loss due to medical conditions such as peptic ulcer disease or malignancy and medications, including ulcer-inducing drugs and those that inhibit marrow function. However, these factors had not been fully accounted for within this study.

The chair stand test, a measure of lower extremity muscle strength, has been demonstrated to be beneficial in determining fall risk [32]. Older persons who had lower chair stand test scores were reported to be associated with a higher risk of fall-related injuries [33]. Moreover, lower extremity weakness was reported to increase the odds of occasional and recurrent falls in older persons [34] since it was associated with abnormal gait, loss of balance, declined mobility, flexibility and functional performance [35]. Besides, strengthening of lower limb muscles has been reported to be effective in preventing falls in older persons [35]. Similarly, we have demonstrated that muscle strength was associated with falls among Malaysian community-dwelling older persons in our earlier pooled data findings [36].

Our study results also showed that the risk of recurrent falls at 18 months was increased among older persons with higher percentage of body fat. One of the probable reasons for this relationship could be due to declined lower extremity muscle strength following excess adiposity, which could affect postural stability and balance [37]. Excess adipose tissue accumulation may also lead to dynapenic obesity, a condition linked to a decline in muscle strength, loss of muscle mass and sarcopenic obesity [38, 39]. Recent studies have also linked adiposity with low-grade inflammation, which not only predisposes individuals to osteoarthritis and dementia but also increases the risk of sarcopenia and osteoporosis [40]. As a result, it may lead to impaired mobility and balance and consequently, increase fall risk in older persons. In an observational study involving 164,737 participants between the ages of 19 to 106 years, older persons with obesity had the odd ratio of 1.10 and 1.12 for one fall and two or more episodes of falls respectively [41].

Increased body fat predisposes individuals to underlying medical conditions such as diabetes, hypertension, heart disease, hypercholesterolemia and stroke. Stroke also has been identified as one of the major risk factors of falls and recurrent falls [42]. Stroke survivors tend to develop fear of falls, which is associated with physical and functional decline, decreased quality of life, impaired social interaction, depression and anxiety [43, 44]. Depressive symptoms and loss of dynamic balance have been demonstrated to increase the risk of falls among stroke survivors [45, 46].

In our longitudinal study, being single (unmarried) was a risk factor of occasional falls during the 18th-months follow-up. Single older persons were commonly identified as living alone, experiencing loneliness, depressive symptoms and poor health [47, 48]. Similarly, these characteristics were associated with increased risk of falls in older persons. Older persons living alone may be having declined physical fitness because of limited participation in physical activity [49, 50]. Subsequently, having an increased risk of fall-related injuries, mortality and morbidity [49, 50].

One of the main limitations of this study is that we obtained retrospective history of falls subjectively. Older persons may have difficulties recalling their fall events in the past 18-months, causing under or over-reporting of the frequency of falls.

## Conclusions

In conclusion, the incidence rates of occasional and recurrent falls in community-dwelling older Malaysian were 8.47 and 3.21 per 100 person-years respectively. Being female, single, having history of falls, higher depression scale, lower hemoglobin levels and lower chair stand test score remained independent predictors of occasional falls. Having history of falls, being a stroke survivor, higher percentage of body fat and lower chair stand test score appeared as recurrent falls predictors in this model. In this study, we identified predictors for prospective falls using a comprehensive, multidomain approach. Further studies are required to determine the value of individualized interventions informed by such an approach to risk factors identification.

## Data Availability

The datasets used and/or analyzed during the current study are available from the corresponding author on reasonable request.

## Abbreviations

BMI: Body Mass Index
HDL: High-density lipoprotein
LRGS-TUA: Long-term Research Grant Scheme – Towards Useful Ageing
LDL: Low-density lipoprotein
MMSE: Mini-Mental State Examination
MoCA: Montreal Cognitive Assessment
TUG: Time Up and Go
WAIS: Wechsler Adult Intelligence Scale
